# Can food parenting practices explain the association between parental education and children’s food intake? The Feel4Diabetes-study

**DOI:** 10.1017/S1368980022000891

**Published:** 2022-10

**Authors:** Paloma Flores-Barrantes, Christina Mavrogianni, Iris Iglesia, Lubna Mahmood, Ruben Willems, Greet Cardon, Flore De Vylder, Stavros Liatis, Konstantinos Makrilakis, Remberto Martinez, Peter Schwarz, Imre Rurik, Emese Antal, Violeta Iotova, Kaloyan Tsochev, Nevena Chakarova, Jemina Kivelä, Katja Wikström, Yannis Manios, Luis A Moreno

**Affiliations:** 1 Growth, Exercise, Nutrition and Development (GENUD) Research Group, University of Zaragoza, Zaragoza, Spain; 2 Instituto Agroalimentario de Aragón (IA2), Instituto De Investigación Sanitaria Aragón (IIS Aragón), Zaragoza, Spain; 3 Department of Nutrition and Dietetics, School of Health Sciences & Education, Harokopio University, Athens, Greece; 4 Red de Salud Materno Infantil y del Desarrollo (SAMID), Instituto de Salud Carlos III, C/ Pedro Cerbuna 12, Madrid 50009, Spain; 5 Department of Public Health and Primary Care, Ghent University, Ghent, Belgium; 6 Department of Movement and Sports Sciences, Ghent University, Ghent, Belgium; 7 National and Kapodistrian University of Athens Medical School, Athens, Greece; 8 Extensive Life Oy, Helsinki, Finland; 9 Department of Prevention and Care of Diabetes, Technical University Dresden, Dresden, Germany; 10 Department of Family and Occupational Medicine, University of Debrecen, Debrecen, Hungary; 11 Hungarian Society of Nutrition, Budapest, Hungary; 12 Department of Paediatrics, Medical University, Varna, Bulgaria; 13 Department of Endocrinology, Medical University of Sofia, Sofia, Bulgaria; 14 Population Health Unit, Finnish Institute for Health and Welfare, Helsinki, Finland; 15 Institute of Agri-food and Life Sciences, Hellenic Mediterranean University Research Centre, Heraklion, Greece; 16 CIBER Fisiopatología de la Obesidad y Nutrición, Instituto de Salud Carlos III, Madrid, Spain

**Keywords:** Mediation analysis, Food parenting practices, Parental educational level, Dietary intake, Children, Europe

## Abstract

**Objective:**

This study aimed to investigate the mediating role of food parenting practices (FPP), including home availability of different types of foods and drinks, parental modelling of fruit intake, permissiveness and the use of food as a reward in the relationship between parental education and dietary intake in European children.

**Design:**

Single mediation analyses were conducted to explore whether FPP explain associations between parents’ educational level and children’s dietary intake measured by a parent-reported FFQ.

**Setting:**

Six European countries.

**Participants:**

Parent–child dyads (*n* 6705, 50·7 % girls, 88·8 % mothers) from the Feel4Diabetes-study.

**Results:**

Children aged 8·15 ± 0·96 years were included. Parental education was associated with children’s higher intake of water, fruits and vegetables and lower intake of sugar-rich foods and savoury snacks. All FPP explained the associations between parental education and dietary intake to a greater or lesser extent. Specifically, home availability of soft drinks explained 59·3 % of the association between parental education and sugar-rich food intake. Home availability of fruits and vegetables was the strongest mediators in the association between parental education and fruit and vegetable consumption (77·3 % and 51·5 %, respectively). Regarding savoury snacks, home availability of salty snacks and soft drinks was the strongest mediators (27·6 % and 20·8 %, respectively).

**Conclusions:**

FPP mediate the associations between parental education and children’s dietary intake. This study highlights the importance of addressing FPP in future interventions targeting low-educated populations.

Childhood obesity is one of the most serious global public health problems in the twenty-first century^(1)^, and socio-economic status (SES) is associated with this condition, since it has been inversely associated with adiposity in high-income countries and directly associated in medium to low-income countries^(2,3)^. Unequal access to healthy foods is one mechanism by which SES influences the diet and health of the population, given that as income drops, energy-dense and nutrient-poor foods become an important source in affordable diets^(4)^. In this sense, among the obesity-related factors^(5)^, dietary behaviour is one of the most relevant due to its strong impact on maintaining energy balance^(6)^.

Significant differences have been found in the consumption of fruits according to SES status, for instance children from lower SES consume less fruit and vegetables (F&V) compared with high SES children, and these differences maintain and even grow over time^(7,8)^. Furthermore, evidence exists confirming that a high SES is associated with healthy eating in youth. For instance, Sandvik *et al.* observed that 34·0 % of children from the highest SES group reported consuming fruit every day compared with 27·6 % of children in the lower SES groups^(9)^. In contrast, low SES has been associated with higher consumption of nutrient-poor foods. Parental education has been identified as one of the best proxy indicators of SES^(10)^ and has been widely used in previous studies^(11,12)^. In this sense, previous studies have found that children whose mothers had a low level of education tend to consume energy-dense food and drinks^(13)^ and those whose parents had higher education levels and the highest household income were more likely to be allocated to the healthy dietary pattern, characterised by the inclusion of low-fat, vitamin-rich and wholegrain foods, among others, and less likely to be allocated to a dietary pattern characterised by high sugar consumption^(14)^.

Parental behaviours or actions performed for child-rearing purposes in the contexts of food and feeding are defined as food parenting practices (FPP)^(15)^. FPP influence children’s dietary intake, as well as home food environment characteristics, such as the home availability of foods or the use of food as a reward, and they vary across SES.

Regarding associations between parental education and FPP, results from a nationally representative sample from the USA also indicated that both education and SES were positively associated with home availability of foods, such as fruits, vegetables and specifically, education was negatively associated with salty snacks and sugary drinks availability at home. On the other hand, income was positively associated with dark green vegetables, low-fat milk products and salty snacks availability at home^(16)^. In addition, a previous study by Campbell *et al*.^(17)^ found that families with higher education levels reported a lower frequency of family meals, whereas lower fresh F&V availability at home was reported by families with lower education levels. Moreover, the accumulation of social vulnerabilities, such as lack of social network and migrant background, has also been associated with a processed dietary pattern^(18)^.

Given that in the last years, parenting styles and specifically food parenting styles have been studied to a greater extent than FPP, it is worth mentioning that general parenting styles can be conceived as more distal, higher-order constructs, whereas parenting practices are more proximal determinants of child behaviour^(19)^. Regarding parenting styles and dietary intake in children, a previous systematic review in pre-school aged children reported that two out of three studies reported that authoritative parenting style was associated with higher intake of F&V and from the two studies that examined associations between parenting styles and unhealthy/non-core foods, no significant associations were observed^(20)^. Also, in a 3-year-longitudinal study in Australian children, 6–9-year-old boys whose mothers reported using the authoritarian style were less likely to consume F&V. In the same study, boys and girls with authoritative and permissive fathers, and girls with authoritative mothers at 4–5 years, were more likely to consume F&V 2 and 4 years later^(21)^.

The relevance of studying these practices lies in the fact that, for example when parents use emotional feeding for a prolonged period of time, children may eventually learn to calm themselves by eating^(22)^, which may increase their future overweight risk. A recent systematic review aimed to conclude that FPP receiving the most attention within prospective studies were generally not associated with children’s weight outcomes over time^(23)^. Nevertheless, several FPP, such as home food availability^(24)^ or parental modelling of food intake^(25)^, are associated with children’s dietary intake.

In this sense, given that previous research has showed significant associations between SES indicators, such as parental education and children’s dietary intake, it is relevant to examine through mediation analyses to what extent FPP explain this relationship. In fact, FPP such as food availability and food accessibility have been previously evaluated as potential mediators of such associations^(26,27)^. Also, a previous systematic review aiming to summarise existing evidence regarding the mediators of socio-economic differences in dietary behaviours among youth at the interpersonal level found that availability at home, accessibility at home, food rules and modelling were consistent mediators of this association^(28)^. Nevertheless, some parenting practices, such as permissiveness and allowance, had not been previously assessed as potential mediators of the associations between SES and children’s dietary intake.

However, to our concern, FPP such as the use of food as a reward have not yet been evaluated as potential mediators. Such information might help construct more effective nutrition interventions aiming to alter children’s eating behaviour and promote healthy eating, particularly in vulnerable and low-SES groups. The understanding of these practices as possible mediators of the previously mentioned associations might be useful for future interventions aiming to improve dietary intake in young children through parental behaviour modifications such as avoiding certain FPP and making efforts to use those known to have a positive impact on their children’s dietary intake. Ultimately, this can result in minimising social inequalities in diet and health^(29)^. Thus, this study aimed to examine the mediating role of FPP in explaining the relationship between parental education and children’s dietary intake.

## Materials and methods

### Study design and setting

This cross-sectional analysis used baseline data from the ‘Families across Europe following a hEalthy Lifestyle FOR Diabetes prevention’ (Feel4Diabetes-study), a cluster-randomised study that included a school- and community-based intervention aiming to promote a healthy lifestyle and tackle obesity and obesity-related metabolic risk factors for the prevention of type 2 diabetes among families from vulnerable groups in six European countries. The recruitment of participants was performed in children of 1st, 2nd and 3rd grade (aged 6–9 years at baseline) and their parent or parents through a standardised, multistage sampling approach. More details on the recruitment strategy can be found in the study of Manios *et al.* (2018)^(30)^. The initial study sample included 11 396 families (12 280 children) and were recruited between January and November 2016 in schools from the participating countries. These countries represented low/middle-income countries (Bulgaria and Hungary), high-income countries (Belgium and Finland) and high-income countries under austerity measures (Greece and Spain). Details of the study protocol have been previously published (https://feel4diabetes-study.eu/)^(30)^.

### Participants

**Fig. 1:**
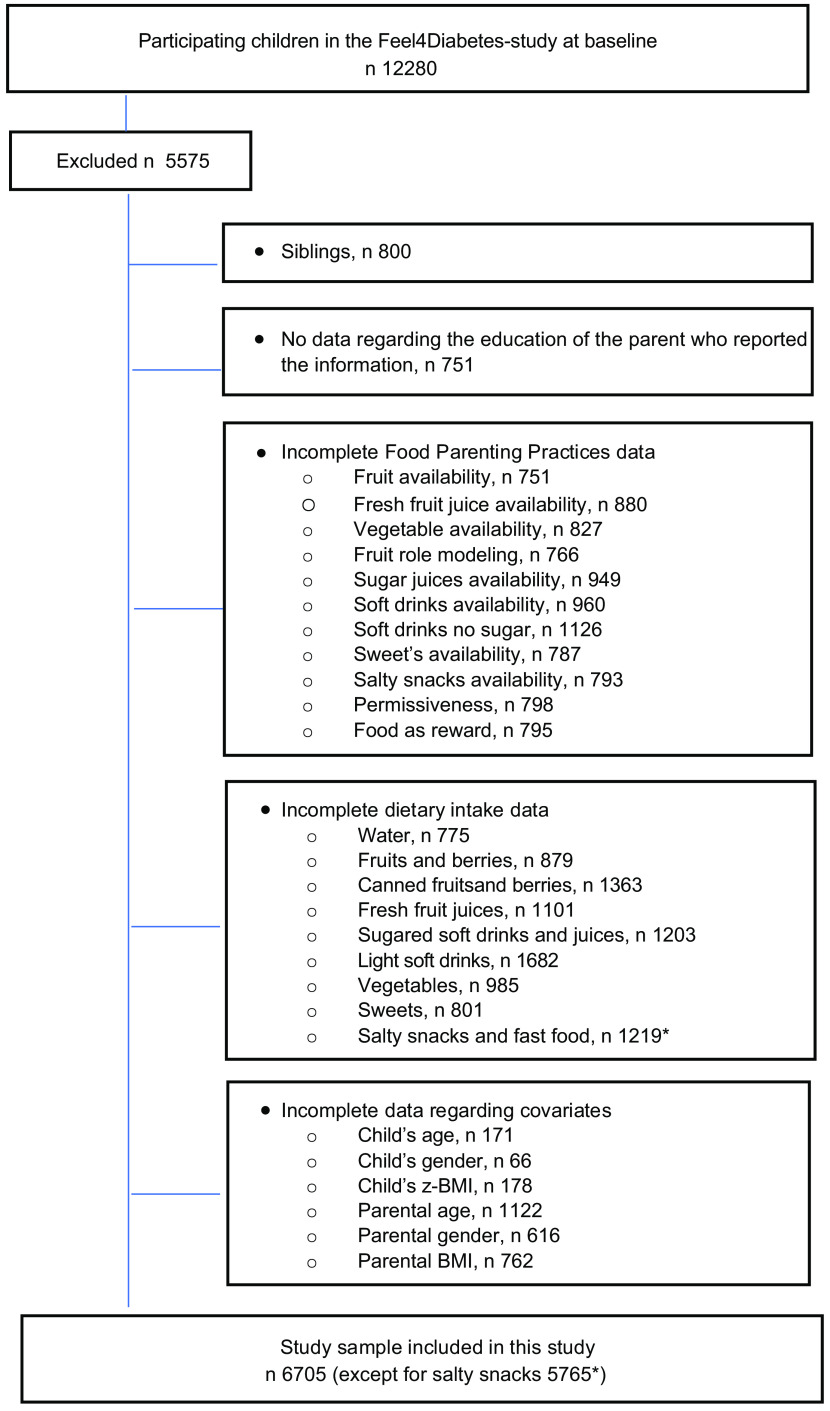
Flow diagram of participant selection

Altogether, 6705 (58·84 %) parent–child dyads (50·7 % girls and 88·8 % mothers) were included from the 11 396 families assessed at baseline. Children with complete information on parental education, children’s food intake, FPP, parental self-reported weight and height and children’s weight and height were included in the present study. In order to avoid duplicate parental information, since some families included more than one child and shared the same reporting parent, we randomly selected one child per family. After this step, 800 children were removed from the main dataset; these represented siblings of participants included in the subsequent analysis. Hence, one child from each family was included and was linked to the reported parental information. A flow diagram of the inclusion of participants is presented in Fig. 1.

Furthermore, to avoid the effect of possible outliers, children consuming more than seven servings per day of F&V were removed from the analyses (*n* 255).

### Measures

One parent per child, either the mother or the father, completed a self-administered questionnaire that assessed socio-demographic characteristics, FPP and their child’s dietary intake, among other energy balance-related behaviours. Anthropometric measurements were conducted according to standardised protocols^(31)^. Children received the parent questionnaire in a closed envelope to take home for completion by one of the parents.

### Parental education level

Education level of both parents was reported by the parent who answered the questionnaire and was asked in a 6-point Likert-type scale question, ranging from ‘less than 6 years’ to ‘more than 16 years’ of education (< 6, 7–9, 10–12, 13–14, 15–16, and > 16). For this study, the education of the reporting parent was considered and dichotomised into ≤ 14 (low-education) and > 14 years (high-education), considering that > 14 years implies attendance of higher education (e.g. a bachelor’s program).

### Dietary intake

Children’s dietary intake was reported by parents with a FFQ, using the question: ‘How often does your child usually consume the following foods and drinks?’, which they could answer by choosing one of the following options: on a weekly (less than 1, 1–2, 3–4 or 5–6 times/week) or daily basis (1–2, 3–4, 5–6 and more than 6 times/d). Beverages assessed were water, fruit juices (freshly squeezed or prepacked without sugar), soft drinks and fruit juices containing sugar and soft drinks without sugar. Foods assessed were fruits and berries (fresh or frozen), fruits and berries (canned), vegetables, sweets and salty snacks and fast food. Intra-class coefficients (ICC) of test–retest showed good reliability of reported food items (ICC = 0·633, (0·371, 0·822)) and have previously been reported in more detail^(32)^. Range categories in times per week (t/w) and times per day (t/d) of the food intake items were recoded to reflect daily intake of servings (s/d) prior to data analyses (less than 1 t/w = 0·14 s/d, 1–2 t/w = 0·21 s/d, 3–4 t/w = 0·5 s/d, 5–6 t/w = 0·79 s/d, 1–2 t/d = 1·5 s/d, 3–4 t/d = 3·5 s/d, 5–6 t/d = 5·5 s/d, and > 6 t/d = 6 s/d).

In this study, four dietary outcomes were assessed: water, F&V, sugar-rich foods and salty snacks and fast food, herein referred to as savoury snacks. Water and savoury snacks were used as single food items, as they were reported. The F&V variable was calculated by summing the total number of daily servings of fruits and berries, canned fruits, 100 % fruit juice and vegetables. The total number of sugar-rich foods was calculated by summing up servings per day of soft drinks and sugary juices and sweets.

### Food parenting practices

The following FPP were included in the questionnaire:

- Home availability of three foods considered to be healthy: fresh fruit, fresh fruit juice and vegetables and home availability of five food items considered to be energy-dense/nutrient-poor: sugary juices, soft drinks, light soft drinks, sweets and salty snacks.
- Parental role modelling of fruit intake: parental consumption of fruit in front of their children.
- Permissiveness: allowance of sweets and salty snacks whenever the child asks for them.
- Use of food as a reward: defined as using sweets, salty snacks or fast food as a reward for their children.

Questions, response options and analytic coding for the analyses are shown in online supplemental Table S1. ICC showed good reliability for home availability of foods (ICC = 0·720 (0·625, 0·794)) and parental role modelling of fruit intake, permissiveness and the use of food as a reward (ICC = 0·695 (0·563, 0·793))^(32)^. Response options on a 5-point Likert scale ranged from ‘very often’ (‘always’ for home food availability) to ‘never’. These categories were reordered to denote increasing use of the practice, from ‘never’ to ‘very often’ (‘always’ for home food availability). To facilitate interpretation, home availability of nutrient-dense foods and parental modelling of fruit intake was classified as positive FPP, while home availability of unhealthy foods, permissiveness of sweets and salty snacks and using food as a reward were classified as negative FPP.

### Covariates: country, age, sex and BMI

Socio-demographic variables included parents’ and children’s age and sex. Children underwent anthropometric measurements that were conducted at school by trained researchers^(31)^ using standard procedures and equipment. Body weight was measured to the nearest 100 g using a portable SECA scale (SECA 213, 214, 217, and 225, Hamburg, Germany). Height was measured to the nearest 0·1 cm using a SECA stadiometer (type SECA 217). Two readings were obtained for each measurement and the mean was used for the analysis. A third measurement was conducted if the previous measurements differed > 100 g for weight and > 1 cm for height. BMI was calculated by dividing weight in kilograms by height in meters squared (as kg/m^2^), and BMI *Z*-scores (*Z*-BMI) were calculated for age and sex according to Cole *et al.*^(33)^. Parental weight (kilograms) and height (meters) were self-reported, and BMI (kg/m^2^) was calculated.

### Data analyses

Normality of the outcome variables was checked with Shapiro–Wilk tests for continuous variables. Given that all continuous variables were not normally distributed, a Mann–Whitney U test was performed for continuous variables for between-group comparisons, while a Pearson’s Chi-square test was used to compare percentages between groups according to SES (Table 1) and sex (see online supplemental Table S2).

**Table 1:** Study participants’ characteristics at baseline by parental education level; *n 6705*

Children	All		Low-education		High-education		*P*
	%	*n*	%	*n*	%	*n*	
Demographics	6705		24·19	1622	75·81	5083	–
Sex							
Girls	50·7	3402	52·9	858	50·0	2544	0·046
Boys	49·3	3303	47·1	764	50·0	2539	
Age (y)							
Mean	8·15		8·23		8·12		<0·001
sd	0·96		0·99		0·95	
Weight (kg)							
Mean	29·57		30·06		29·41		0·008
sd	7·11		7·54		6·96	
Height (cm)							
Mean	130·45		130·13		130·55		0·146
sd	7·88		7·91		7·87	
BMI (kg/m^2^)							
Mean	17·20		17·57		17·08		<0·001
sd	2·79		3·09		2·68	
*Z*-BMI							
Mean	0·54		0·65		0·51		<0·001
sd	1·07		1·15		1·04	
Children’s dietary intake, servings/d							
Water							
Mean	3·83		3·93		3·80		0·001
sd	1·73		1·79		1·72	
Fruits & vegetables							
Mean	2·91		2·77		2·95		<0·001
sd	1·38		1·47		1·34	
Sugar-rich foods							
Mean	1·21		1·50		1·12		<0·001
sd	1·18		1·64		0·97	
Savoury snacks							
Mean	0·34		0·45		0·29		<0·001
sd	0·48		0·67		0·38	
Reporting parents							
Demographics	6705		24·19	1622	75·81	5083	–
Sex							
Mothers	88·8	5952	85·3	1383	89·9	4569	
Fathers	11·2	753	14·7	239	10·1	514	<0·001
Country							
Belgium	17·9	1200	16·0	259	18·5	941	<0·001
Bulgaria	19·3	1295	17·4	282	19·9	1013	
Finland	13·8	928	5·4	88	16·5	837	
Greece	20·8	1392	33·6	545	16·7	847	
Hungary	14·7	983	26·4	429	10·9	554	
Spain	13·6	910	1·2	19	17·5	891	
Age (years)							
Mean	38·54		37·21		38·96		<0·001
sd	5·09		5·85		4·75	
BMI (kg/m^2^)							
Mean	24·40		24·97		24·21		<0·001
sd	4·54		4·94		4·39	

SES, socio-economic status, *Z*-BMI, BMI *Z* score according to Cole *et al*. (2012).

*n* 6705, except for salty snacks, *n* 5765. Boldface indicates statistical significance between SES at *P* < 0·05.

Chi-square test was used to test differences by SES for categorical data.

Mann–Whitney *U* tests were performed to test differences by education in log-transformed continuous variables.

Low-education was defined as <14 years of parental education and high-education was defined as > 14 years of parental education.

Fruits and vegetables: fresh or frozen fruit and berries, canned fruit, fresh fruit juices and vegetables.

Sugar-rich foods: Sugar-sweetened beverages (sugar juices and soft drinks) and sweets.

Savoury snacks: salty snacks and fast food (e.g. one small hamburger, one small bag of chips, one slice of pizza).

As the four dietary outcome variables were not normally distributed, the data were log-transformed for the analyses (ln (x + 10). For interpretation purposes, results describing the association between SES and dietary intake of foods and beverages are also reported using the non-transformed data (normal scale).

Mediation models are used to examine the possible causal processes through which a predictor leads to an outcome^(34)^. To investigate whether FPP mediated the associations between parental education and children’s food intake, Baron & Kenny’s four-step approach for mediation analyses was used^(35)^. First, to determine whether FPP were significant mediators of the relationship between parental education and children’s dietary intake, it was necessary to show that: (i) the predictor (parental education) is associated with the mediator (FPP); and (ii) the mediator (FPP) is associated with the outcome (children’s dietary intake) while simultaneously controlling for the tested predictor (parental education). These facts explain the reason for running adjusted linear regressions to examine the associations between parental education and the potential mediators (FPP) and between children’s dietary intake and the FPP (Table 2). Then, based on the hypothesis that FPP could mediate the associations between parental education and dietary intake, the mediation models were examined using the PROCESS macro 3.1.4 software for SPSS by Andrew Hayes^(36)^. In PROCESS, Model 4 software was applied for simple mediations

**Table 2:** Associations between parental education, food parenting practices and dietary intake in children, *n* 6705

Food parenting practices	Parental education			Water			Fruits and vegetables			Sugar-rich foods			Savoury snacks		
	Adj. *R*^2^	*β*	*P* value	Adj. *R*^2^	*β*	*P* value	Adj. *R*^2^	*β*	*P* value	Adj. *R*^2^	*β*	*P* value	Adj. *R*^2^	*β*	*P* value
Positive food parenting practices															
HA fruit	0·082	0·144	<0·001	0·188	0·057	<0·001	0·120	0·290	<0·001	0·147	−0·046	<0·001	0·128	−0·088	<0·001
HA fresh fruit juice	0·123	0·028	0·026	0·185	−0·010	0·417	0·094	0·240	<0·001	0·149	−0·060	<0·001	0·121	−0·025	0·061
HA vegetables	0·121	0·097	<0·001	0·188	0·055	<0·001	0·112	0·281	<0·001	0·146	−0·026	0·030	0·124	−0·063	<0·001
Parental modelling of fruit intake	0·041	0·026	0·044	0·193	0·094	<0·001	0·191	0·393	<0·001	0·152	−0·080	<0·001	0·125	−0·064	<0·001
Negative food parenting practices															
HA sugar juices	0·071	−0·088	<0·001	0·191	−0·079	<0·001	0·044	−0·039	0·001	0·201	0·243	<0·001	0·141	0·148	<0·001
HA soft drinks	0·212	−0·122	<0·001	0·192	−0·097	<0·001	0·050	−0·095	<0·001	0·197	0·257	<0·001	0·152	0·202	<0·001
HA light soft drinks	0·184	0·003	0·813	0·186	−0·034	0·005	0·043	0·015	0·254	0·147	0·038	0·002	0·123	0·050	<0·001
HA sweets	0·195	0·007	0·568	0·189	−0·059	<0·001	0·048	−0·084	<0·001	0·267	0·388	<0·001	0·148	0·186	<0·001
HA salty snacks	0·233	−0·079	<0·001	−0·186	−0·043	0·001	0·047	−0·077	<0·001	0·199	0·263	<0·001	0·245	0·410	<0·001
Permissiveness	0·084	−0·078	<0·001	−0·192	−0·089	<0·001	0·051	−0·093	<0·001	0·229	0·302	<0·001	0·182	0·258	<0·001
Using food as a reward*	0·072	−0·099	<0·001	0·189	−0·063	<0·001	0·045	−0·050	<0·001	0·182	0·198	<0·001	0·159	0·202	<0·001
Children’s food intake															
Water	0·185	0·041	0·001	–	–		–	–		–	–		–	–	
Fruit and vegetables	0·043	0·053	<0·001	–	–		–	–		–	–		–	–	
Sugar-rich foods	0·146	−0·052	<0·001	–	–		–	–		–	–		–	–	
Salty snacks and fast food	0·121	−0·123	<0·001	–	–		–	–		–	–		–	–	

*β* , Standardised coefficients; HA, home availability.

* *n* 6705, except for salty snacks and fast food (savoury snacks), *n* 5765.

Individual linear regressions were performed using the log-transformed scales of outcome variables and were adjusted for country and parental and children’s sex, age and BMI.

Parental education was also included as covariate to test the associations between FPP and dietary intake.

Boldface indicates statistical significance.

**Fig. 2:**
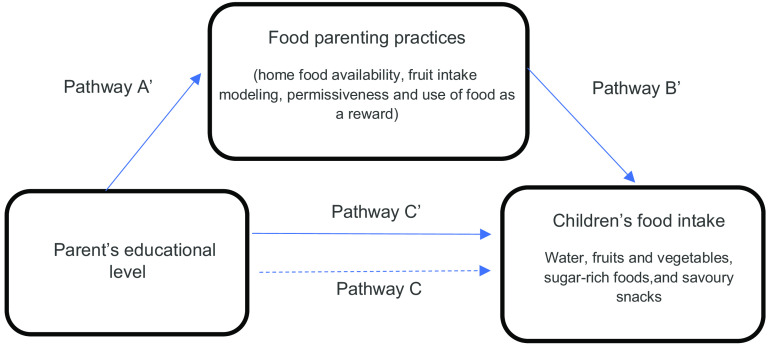
Graphical illustration of the possible interactions between parent’s educational level, FPP and children’s food intake. Simple mediation analyses adjusted by country, parental age, BMI and sex, and children’s age, BMI and sex. Pathway A’: Association between parent’s educational level and FPP. Pathway B’: Association between FPP and children’s food intake. Pathway C’: Direct association between parent’s educational level and children’s food intake after adjustment for each mediator (FPP). Pathway C: The total effect (C) shows the association between parent’s educational level and dietary intake. A * B: Indirect effect of each FPP on the association between parent’s educational level and dietary intake. Abbreviations: FPP, food parenting practices

Mediation models were performed individually to examine the mediating role of each FPP on the association between parental education and the four outcomes. As shown in Fig. 2, the independent variable was parental education, dependent variables were children’s food intake and the potential mediator variables were the positive (e.g. home availability of fruit) and negative (e.g. permissiveness) FPP. Included covariates were country; parental age, sex and BMI; and children’s age, sex and BMI. In the model, the associations between parental education and FPP (a’ – coefficient), FPP and dietary outcomes (b’ – coefficient) and parental education and dietary outcomes (c’ – coefficient) are illustrated. The indirect effects (A * B) data, which is obtained by multiplying a’ x b’ coefficient, are generated with 95 % CI, representing *P*-values < 0·05 rather than generating exact *P*-values. Significant mediations were then calculated as percentages by dividing A * B by the total effect (c – coefficient).

Descriptive and mediation analyses were completed using IBM SPSS Statistics (IBM SPSS Statistics for Windows, Version 26.0. IBM Corp.).

## Results

### Participant characteristics

In total, 6705 child–parent dyads from the six participating countries from the Feel4Diabetes-study were included in this study. Table 1 shows the characteristics of the children (50·7 % girls) and the parents (88·8 % mothers) by parental education group. The mean age of the children was 8·15 ± 0·96 years. Parents’ mean age was 38·54 ± 5·09 years. Of all participating parents, 75·8 % stated that they had completed 14 years of education or more. Regarding dietary intake, children whose parents had a low education level had higher consumption of water, sugar-rich foods and savoury snacks than children from parents with high education level. Conversely, the consumption of F&V was significantly higher in the high-education level group. The description of participants’ characteristics by sex is shown in online Supplemental Table S2.

### Associations between SES, children’s dietary intake and FPP

First, the associations between parental education, FPP and children’s food intake adjusting for covariates were assessed (Table 2). Parental education was associated with higher water and F&V intake and with lower sugar-rich food and savoury snack consumption.

Parental education was associated with nine of the eleven FPP. No associations were observed between parental education and home availability of light soft drinks or sweets, and these FPP were therefore not considered for subsequent analyses.

Out of a total of fourty-four associations between FPP and children’s food intake, fourty-one were significant. Regarding FPP and children’s food intake, several direct significant associations were observed between positive FPP, like modelling of fruit intake, and water and F&V intake. Conversely, several inverse significant associations were observed between positive FPP and energy-dense/nutrient-poor foods. Nevertheless, no associations were found between the FPP of home availability of fresh fruit juice and water or savoury snack intake or the FPP of home availability of light soft drinks and F&V.

### Mediating effect of FPP on the associations between SES and children’s dietary intake

**Fig. 3:**
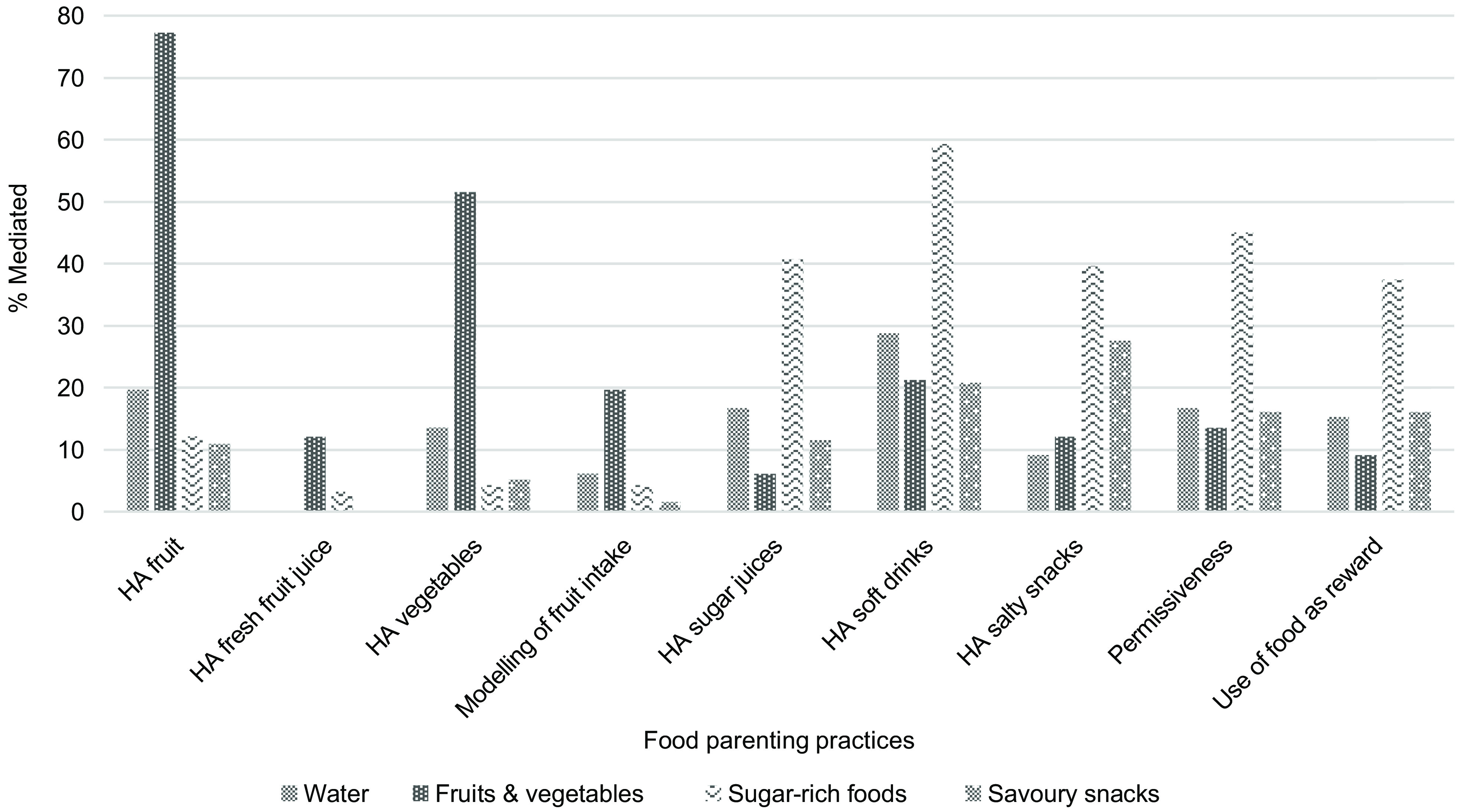
Mediating effect of FPP on the association between parental education and dietary intake of water, fruits & vegetables, sugar-rich foods, and savoury snacks. HA, home availability

The potential mediating effect of positive and negative FPP in the association between parental education and children’s dietary intake of water, F&V, sugar-rich foods and savoury snacks was evaluated while adjusting for covariates. To facilitate interpretation of the models, a graphical illustration of the mediation pathways between parental education, FPP and dietary intake is shown in Fig. 2 and another to illustrate the proportions mediated by each FPP in Fig. 3.

Home availability of fruits and soft drinks appeared to be the most important mediator explaining the association between parental education and children’s water intake (Table 3). It is worth pointing out that the other FPP assessed were also found to be significant mediators but to a lesser extent.

**Table 3:** Total associations (c)* direct associations (c’) and indirect effects between parental education and water intake adjusted for significant mediators

Food parenting practices	Direct effect C’ path				Indirect effect A * B path				
	Log-scale†		Normal – scale‡		Log-scale		Normal – scale	
	*β*	95 % CI	*β*	95 % CI	*β*	95 % CI	*β*	95 % CI	Mediation %
Positive FPP									
HA fruit	0·053	0·015, 0·091	0·042	0·053, 0·136	0·013	0·007, 0·021	0·022	0·008, 0·037	19·7
HA fresh fruit juice	0·066	0·028, 0·104	0·064	–0·030, 0·158	0·000	–0·002, 0·001	0·001	–0·002, 0·004	–
HA vegetables	0·057	0·019, 0·095	0·045	–0·049, 0·139	0·009	0·004, 0·014	0·019	0·009, 0·032	13·6
Modelling of fruit intake	0·062	0·024, 0·100	0·054	–0·039, 0·148	0·004	0·000, 0·008	0·010	0·000, 0·020	6·1
Negative FPP									
HA sugar juices	0·055	0·017, 0·092	0·039	–0·055, 0·132	0·011	0·007, 0·016	0·025	0·016, 0·036	16·7
HA soft drinks	0·047	0·009, 0·085	0·022	–0·072, 0·117	0·019	0·013, 0·026	0·042	0·028, 0·057	28·8
HA salty snacks	0·060	0·022, 0·098	0·055	–0·039, 0·149	0·006	0·002, 0·009	0·009	0·001, 0·018	9·1
Permissiveness	0·055	0·017, 0·092	0·041	–0·052, 0·135	0·011	0·007, 0·016	0·023	0·013, 0·034	16·7
Use of food as reward	0·056	0·018, 0·094	0·043	–0·051, 0·137	0·010	0·006, 0·015	0·021	0·011, 0·033	15·2

FPP, food parenting practices; HA, home availability.

*n* 6705.

Single mediation analyses were adjusted for country and parental and children’s sex, age, and BMI.

* Total effect (C pathway) of the association between parental education and water intake: *β*_log_ = 0·066 (0·028, 0 104); *β*_normal_ = 0·064 (-0·030, 0 158).

† Results on the log-scale were used for determining statistical significance.

‡ Results on the normal scale (servings/d) are used for interpretation purposes only.

For the association between parental education and children’s F&V intake (Table 4), home availability of fruits, vegetables and soft drinks and parental modelling of fruit intake were found to be significant mediators, explaining 77·3 %, 51·5 %, 21·8 % and 19·7 % of this association, respectively.

**Table 4:** Total associations (c)*, direct associations (c’) and indirect effects between parental education and fruit and vegetable intake adjusted for significant mediators

Food parenting practices	Direct effect C’ path				Indirect effect A * B path				
	Log-scale†		Normal – scale‡		Log-scale		Normal – scale	
	*β*	95 % CI	*β*	95 % CI	*β*	95 % CI	*β*	95 % CI	Mediation %
Positive FPP									
HA Fruit	0·014	−0·016, 0·045	−0·007	−0·087, 0·073	0·051	0·041, 0·063	0·114	0·090, 0·139	77·3
HA Fresh fruit juice	0·058	0·027, 0·088	0·085	0·006, 0·165	0·008	0·001, 0·015	0·022	0·004, 0·041	12·1
HA Vegetables	0·032	0·002, 0·062	0·031	−0·049, 0·111	0·034	0·024, 0·044	0·077	0·054, 0·099	51·5
Modelling of fruit intake	0·053	0·025, 0·082	0·077	0·000, 0·153	0·013	0·001, 0·025	0·031	0·001, 0·060	19·7
Negative FPP									
HA Sugar juices	0·062	0·030, 0·093	0·098	0·016, 0·181	0·004	0·002, 0·007	0·009	0·002, 0·017	6·1
HA Soft drinks	0·052	0·020, 0·083	0·073	−0·010, 0·155	0·014	0·010, 0·020	0·035	0·023, 0·047	21·2
HA Salty snacks	0·058	0·027, 0·090	0·088	0·006, 0·170	0·008	0·004, 0·011	0·019	0·011, 0·029	12·1
Permissiveness	0·057	0·026, 0·088	0·088	0·006, 0·170	0·009	0·005, 0·013	0·019	0·011, 0·029	13·6
Use of food as reward	0·060	0·028, 0·091	0·095	0·013, 0·177	0·006	0·003, 0·010	0·012	0·004, 0·021	9·1

FPP, food parenting practices; HA, home availability.

*n* 6705.

Single mediation analyses were adjusted for country and parental and children’s sex, age and BMI.

* Total effect (C pathway) of the association between parental education and F&V intake: *β*_log_ = 0·066, 95 % CI (0·035, 0·097); *β*_normal_ = 0·107 (0·025, 0 189).

† Results on the log-scale were used for determining statistical significance.

‡ Results on the normal scale (servings/d) are used for interpretation purposes only.

Regarding the association between parental education and children’s sugar-dense food intake (Table 5), all FPP assessed were found to be significant mediators. However, the most significant mediators were found to be negative FPP, particularly home availability of soft drinks and permissiveness, for which proportions mediated were 59·3 and 45·1 %, respectively.

**Table 5:** Total associations (c)*, direct associations (c’) and indirect effects between parental education and sugar-rich foods intake adjusted for significant mediators

Food parenting practices	Direct effect C’ path				Indirect effect A * B path				
	Log-scale†		Normal – scale‡		Log-scale		Normal – scale	
	*β*	95 % CI	*β*	95 % CI	*β*	95 % CI	*β*	95 % CI	Mediation %
Positive FPP									
HA fruit	−0·079	−0·121, −0·038	−0·226	−0·293, −0·158	−0·011	−0·018, −0·005	−0·024	−0·036, −0·013	12·1
HA fresh fruit juice	−0·088	−0·129, −0·047	−0·245	−0·312, −0·179	−0·003	−0·006, −0·000	−0·004	−0·009, −0·001	3·3
HA vegetables	−0·086	−0·128, −0·045	−0·240	−0·307, −0·173	−0·004	−0·009, 0·000	−0·010	−0·019, −0·001	4·4
Modelling of fruit intake	−0·087	−0·128, −0·046	−0·244	−0·311, −0·178	−0·004	−0·007, −0·000	−0·005	−0·010, 0·000	4·4
Negative FPP									
HA sugar juices	−0·054	−0·094, −0·014	−0·201	−0·267, −0·136	−0·037	−0·048, −0·027	−0·048	−0·063, −0·034	40·7
HA soft drinks	−0·037	−0·077, 0·004	−0·161	−0·226, −0·096	−0·054	–0·067, –0·043	−0·088	−0·109, −0·069	59·3
HA salty snacks	−0·055	−0·095, −0·015	−0·199	−0·265, −0·134	−0·036	−0·047, −0·025	−0·050	−0·067, −0·035	39·6
Permissiveness	−0·050	−0·089, −0·011	−0·192	−0·256, −0·127	−0·041	−0·054, −0·027	−0·058	−0·078, −0·038	45·1
Use of food as reward	−0·057	−0·098, −0·016	−0·194	−0·259, −0·128	−0·034	−0·045, −0·024	−0·056	−0·075, −0·039	37·4

FPP, food parenting practices; HA, home availability.

*n* 6705.

Single mediation analyses were adjusted for country and parental and children’s sex, age and BMI.

Sugar-rich foods included the sum of sweets (e.g. one chocolate bar or half a cup of sweets, cookies or icecream) and soft drinks (e.g. one glass of one cup of soft drinks and juices containing sugar).

* Total effect (C pathway) of the association between parental education and sugar-rich foods intake: *β*_log_= −0·091, 95 % CI (−0·132, -0·050); *β*_normal_ = −0·249 (−0·316, −0·183).

† Results on the log-scale were used for determining statistical significance.

‡ Results on the normal scale (servings/d) are used for interpretation purposes only.

The association between parental education and savoury snack intake (Table 6) was explained by home availability of salty snacks (27·6 %) and home availability of soft drinks (20·8 %) as well as the other FPP assessed, which also proved to be significant mediators, but to a lesser extent.

**Table 6:** Total associations (c)*, direct associations (c’) and indirect effects between parental education and savoury snacks adjusted for significant mediators

Food parenting practices	Direct effect C’ path				Indirect effect A * B path				
	Log-scale†		Normal – scale‡		Log-scale		Normal – scale	
	*β*	95 % CI	*β*	95 % CI	*β*	95 % CI	*β*	95 % CI	Mediation %
Positive FPP									
HA fruit	−0·170	−0·211, −0·130	−0·107	−0·136, −0·079	−0·021	−0·029, −0·014	−0·010	−0·015, −0·005	10·9
HA fresh fruit juice	−0·191	−0·231, −0·151	−0·117	−0·145, −0·089	−0·001	−0·003, 0·000	−0·001	−0·002, 0·000	–
HA vegetables	−0·181	−0·222, −0·141	−0·111	−0·140, −0·083	−0·010	−0·016, −0·005	−0·006	−0·011, −0·002	5·2
Modelling of fruit intake	−0·188	−0·228, −0·148	−0·116	−0·144, −0·088	−0·003	−0·006, −0·000	−0·001	−0·003, 0·000	1·6
Negative FPP									
HA sugar juices	−0·169	−0·209, −0·129	−0·105	−0·133, −0·077	−0·022	−0·030, −0·016	−0·012	−0·017, −0·008	11·5
HA soft drinks	−0·152	−0·192, −0·112	−0·094	−0·122, −0·065	−0·040	−0·050, −0·030	−0·024	−0·031, 0·017	20·8
HA salty snacks	−0·138	−0·176, −0·101	−0·091	− 0·118, −0·064	−0·053	−0·069, −0·037	−0·027	-0·035, −0·019	27·6
Permissiveness	−0·160	−0·199, −0·122	−0·100	−0·127, −0·072	−0·031	−0·043, −0·021	−0·18	−0·024, −0·011	16·1
Use of food as reward	−0·161	−0·200, −0·122	−0·098	−0·126, −0·070	−0·031	−0·041, −0·021	−0·019	−0·027, −0·012	16·1

FPP, food parenting practices; HA, home availability.

*n* 5765.

Single mediation analyses were adjusted for country and parental and children’s sex, age and BMI.

Savoury snacks include salty snacks and fast-food items like one small hamburger, one bag of chips or one slice of pizza.

* Total effect (C pathway) of the association between SES and savoury snacks intake: *β*_log_= −0·192, 95 % CI (−0·232, −0·152); *β*_normal_ = −0·117 (−0·146, −0·089).

† Results on the log-scale were used for determining statistical significance.

‡ £ Results on the normal scale (servings/d) are used for interpretation purposes only.

## Discussion

The current study shows that inequalities in food intake according to parental education were partly mediated by the addressed FPP in European children from the Feel4Diabetes-study. In fact, almost all of them appeared to explain the associations to a greater or lesser extent. Given that parental education is difficult to modify in the short term, FPP appear to be interesting factors for potential modification.

Intake of assessed food items was significantly affected by parental education for all items except water (see online supplemental Table S3), being directly associated with water, F&V and inversely associated with sugar-rich foods and savoury snacks; nevertheless, water intake was used in later analyses because of the observed associations in the adjusted regression models. An explanation for this may be that the assessment method for water intake was not ideal, since repeated 24-h recalls or specific tools are preferable to FFQ for water intake^(37)^. Parents may have found it difficult to estimate an average daily consumption since water intake is usually distributed throughout the day and might be difficult to quantify properly. Also, water intake might not strictly depend on parental education, since all the families from our study have drinking water access, and water was therefore available in every household.

No associations were observed between parental education and home availability of light soft drinks or sweets; therefore, these FPP were not considered for subsequent mediation analyses. Nevertheless, results from the adjusted linear regressions showed that home availability of light soft drinks was inversely associated with water intake, indicating that the presence of these beverages might reduce the amount of water intake. These associations are in line with the findings of Galastri *et al*.^(38)^, which aimed to assess the association between ultra-processed food consumption and total water intake in a national representative sample from the USA, indicated that the consumption of artificially sweetened beverages was associated with a reduction in water consumption. On the other hand, home availability of light soft drinks was directly associated with consumption of sugar-rich foods and savoury snacks, indicating that even though light soft drinks might not be a substantial source of calories, their availability at home may have an association with a pattern of high consumption of energy-dense foods such as savoury snacks.

In our study, permissiveness and home availability of both sugary juices and soft drinks were inversely associated with water intake. In a previous study in 6- to 8-year-old European children that aimed to evaluate the associations between parenting practices towards fruit juices and soft drinks and water consumption of children, children’s water intake was found to be favourably influenced by less parental allowance, low home availability and high parental self-efficacy in managing intake^(39)^. On this basis, we assessed the mediation effect of FPP on the association between parental education and water intake and found that home availability of soft drinks was the strongest mediator. This finding indicates that the association between parental education and water consumption is significantly explained by the presence of soft drinks at home, which may replace water intake in children. Interestingly, fresh fruit juice was not a significant mediator, indicating that the consumption of water is not affected or replaced by that of fresh fruit juice. As shown in our study, the physical presence of sugar-sweetened beverages at home is inversely associated with water intake; however, as shown in a randomised controlled trial aiming to decrease sugar-sweetened beverages intake in Dutch adolescents^(40)^, the decrease in sugar-sweetened beverages consumption over time does not necessarily lead to an increase in water consumption. This indicates that water intake promotion may be necessary to achieve the corresponding recommendations.

In a previous study in 11-year-old Dutch children, that evaluated the potential mediating effect of home environment characteristics in the association between maternal educational level and children’s healthy eating behaviour, results indicated that home availability of fruit significantly mediated this association. In fact, home availability appeared to be a significant mediator when evaluated separately and in combination with other factors, such as parental fruit intake and fruit consumption rules^(41)^. It is worth mentioning that parental intake of fruit refers to their diet and differs from parental modelling of fruit intake, which is often used to refer to more intentional efforts made by parents to actively demonstrate healthy eating for the child^(15)^.

In our study, important significant mediators on the association between parental education and sugar-rich food intake were primarily those considered as negative FPP, specifically, home availability of soft drinks and permissiveness. As observed by Robert *et al*.^(42)^, parents with the highest mean scores for permissive parenting frequently used rewards, defined as the use of both tangible items and food to reward children for eating and behaviours. It was also found in the current study that the use of food as a reward was significantly correlated with permissiveness (data not shown), indicating that these practices may be used in combination. Moreover, the link between parental education and the use of food as a reward could be explained by the fact that food may be recognised as an easy and affordable reward that will be accepted by the child.

A previous study in 3- to 11-year-old Australian children found that covert control feeding strategies, defined as the way in which parents promote the consumption of healthy food by managing the child’s environment by providing primarily healthy foods, were significantly associated with lower unhealthy snack intake but not with healthy snack food intake by children^(43)^, indicating a positive effect of healthy home food availability in terms of lower unhealthy snack intake over time.

Other FPP, such as food accessibility, have been explored for their potential role as mediators in the association between SES and dietary intake. For instance, a previous study in Norwegian adolescents showed that food accessibility and perceived rules were significant mediators of the associations between parental education and soft drink consumption^(27)^, indicating that other FPP besides the ones we explored may also play an important role.

An option for replacing the use of food as a reward and its negative effects on diet, and consequently on health, would be the use of social rewards, which are inexpensive or free and can be even more powerful than material rewards. Examples of social rewards include those characterised by affection, such as hugs and smiles, and those including attention and activities, such as playing the child’s favourite game together, reading a story or encouraging them to help with home tasks like preparing dinner^(44)^. Permissiveness regarding food intake might be accompanied by permissiveness in other aspects of the child’s activities; in this sense, it could be interesting to evaluate if permissiveness is also applied to other aspects of life, such as physical activity and sedentary behaviours like the use of screens.

Humans learn by imitation and reference^(45)^; therefore, it is also possible that parents, regardless of their educational level, were raised surrounded by the same FPP they use. This means that they might have inherited lifestyle habits and family rules and they use these with their own children. At this point, it is important to break the cycle, so they recognise their behaviours and can make efforts to improve.

A major strength of our study is the large and pan-European sample and the standardisation of measurements, which was followed across all centres. Also, to our knowledge, this is the first study examining the mediating role of permissiveness and the use of food as a reward explaining differences in European children’s dietary intake by SES. However, our study has several limitations. Firstly, a FFQ was used to assess regular dietary intake, which may have introduced self-report bias, but this weakness is very hard to overcome when studying food intake^(46)^. In addition, parents reporting both food intake and FPP might overestimate the association between the variables. However, as some of the children were only first graders, it was not possible to get self-reported food intake data from the children. In this study, education was used as the main determinant of dietary intake; nevertheless, it could be important to include other SES variables besides education, such as occupation^(47)^, employment status or income or even composite indices^(48)^, considering that each of them should be chosen according to their strengths, limitations and depending on the sample‘s characteristics. We selected the education of the reporting parent as the exposure variable since we considered that it was relevant to consider how education reflects on parents’ behaviour and whether it determines if they are more permissive or tend to use food as a reward with their children. Nevertheless, for future studies, it might be important to consider the educational level and FPP of both parents and, for example, to evaluate whether they are consistent with each other or contrary in some respects.

A recent study that aimed to evaluate the associations between parents’ work status and the dietary consumption patterns of Australian pre-school children^(49)^ found that depending on the work status and educational level attained by mothers or fathers, children presented significant differences in terms of F&V, high-fat foods and high-sugar foods consumption. This indicates that not only education but also work status has an important role in determining the dietary intake of children at this age.

Even though there was an initial sample loss of 41·2 %, the proportion of low-educated and high-educated parents was very similar in the included sample and in the excluded sample, which indicates that no selection bias might have occur.

Parents should be aware that there are modifiable practices that they can use in the home food environment, such as home availability, and they can try to enhance these to improve their children’s diet. There is a need for family-focused research that identifies social aspects of the home environment that potentially impact on dietary intake of children^(50)^. It remains important to encourage parents to understand the importance of avoiding negative FPP.

In conclusion, this study highlighted the role of FPP in explaining the associations between parental education and children’s intake of water, fruits and vegetables, sugar-rich foods and savoury snacks. Encouraging parents, especially those with a low level of education, to increase the use of positive FPP, such as modelling of fruit intake, and to avoid the use of negative FPP, such as home availability of soft drinks, might help to tackle health and dietary inequalities by improving children’s intake of these food groups. Health professionals should understand not only the challenges but also the opportunities and possibilities that parents can have if they improve the FPP they use. These findings may broaden the understanding of potential pathways by which various factors might influence children’s dietary intake, helping researchers to better design nutrition-focused interventions.

## Supplementary material

For supplementary material accompanying this paper visit https://doi.org/10.1017/S1368980022000891. click here to view supplementary material
