# Supplementary material for: Can food parenting practices explain the association between parental education and children’s food intake? The Feel4Diabetes-study
Source: Public Health Nutr. 2022 Apr 13;25(10):2758–71. doi: 10.1017/S1368980022000891 (PMC9991856; doi:10.1017/S1368980022000891)
Supplement: Supplementary file 1 [file S1368980022000891sup001.docx]

**Supplementary**

| **Table S1.** Questions, response options and analytic coding for the variables used in the analyses. The Feel4Diabetes study | | |
| --- | --- | --- |
| **Questionnaire item** | **Response options** | **Analytic coding** |
| **Children’s dietary intake** | | |
| **Please, indicate how often does your child regularly consume the following foods and drinks.** | | |
| 1. Water (1 glass or 1 cup) | 8 response options:   1. Less than once per week 2. 1 to 2 times per week 3. 3-4 times per week 4. 5-6 times per week 5. 1-2 times per day 6. 3-4 times per day 7. 5-6 times per day 8. More than 6 times per day | A continuous variable indicating servings per day was created according to the frequency of intake   1. Less than 1 t/w = 0.14 s/d 2. 1-2 times per week = 0.21 s/d 3. 3-4 t/w = 0.5 s/d 4. 5-6 t/w = 0.79 s/d 5. 1-2 t/d = 1.5 s/d 6. 3-4 t/d = 3.5 s/d 7. 5-6 t/d = 5.5 s/d 8. >6 t/d = 6 s/d. |
| 1. Fruits and berries, fresh or frozen (1 tennis ball sized fruit or 2 small fruits or half a cup of chopped) |  |  |
| 1. Fruits and berries, canned (half a cup) or dried (a quarter of cup) |  |  |
| 1. Fruit juices, freshly squeezed or prepacked without sugar (1 glass or 1 cup) |  |  |
| 1. Soft drinks and juices containing sugar (1 glass or 1 cup) |  |  |
| 1. Soft drinks, diet (light) (1 glass or 1 cup) |  |  |
| 1. Vegetables (half a cup or at the size of a tennis ball of tomato, broccoli, leafy vegetables etc.) |  |  |
| 1. Sweets (1 chocolate bar or half a cup of sweets, cookies, or ice-cream) |  |  |
| 1. Salty snacks/fast food (e.g.,1 small hamburger, 1 small bag of chips, 1 slice of pizza) |  |  |
| **Food parenting practices** | | |
| **On a weekly basis**, **how frequently are the following food item available in your home?** | | |
| 1. Fruits | 5 response options:   1. Always 2. Often 3. Sometimes 4. Rarely 5. Never | Categories were recoded from never to always:   1. Never 2. Rarely 3. Sometimes 4. Often 5. Always |
| 1. Fruit juices, freshly - squeezed or prepacked without sugar |  |  |
| 1. Fruit juices, prepacked, containing sugar |  |  |
| 1. Soft drinks containing sugar |  |  |
| 1. Soft drinks without sugar |  |  |
| 1. Vegetables |  |  |
| 1. Sweets, biscuits, ice cream, cakes, pastries |  |  |
| 1. Salty snacks (e.g., chips, savoury pastries) |  |  |
| **On a weekly basis, how often do you use the following practices:** | | |
| 1. How often do you consume fresh fruits with your child? | 1. Very often 2. Often 3. Sometimes 4. Rarely 5. Never | Categories were recoded from never to very often:   1. Never 2. Rarely 3. Sometimes 4. Often 5. Very often |
| 1. How often do you reward your child with sweets, salty snacks (e.g. potato chips) or fast food |  |  |
| 1. How often do you allow your child to eat sweets and/or salty snacks whenever he/she asks for |  |  |
|  | | |

| **Table S2.** Study participants’ characteristics at baseline by sex; *n= 6705* | | | | |
| --- | --- | --- | --- | --- |
| **Children** | **All** | **Girls** | **Boys** | ***p*** |
| **Demographics**  % (n)  **SES,** % (n)  Low-SES  High-SES  **Country,** % (n)  Belgium  Bulgaria  Finland  Greece  Hungary  Spain  **Age (y)**, mean ± SD  **Weight (Kg)**, mean ± SD  **Height (cm)**, mean ± SD  **BMI (Kg/m^2^),** mean ± SD  **z-BMI**, mean ± SD | 6705  24.19 (1622)  75.81 (5083)  17.9 (1200)  19.3 (1295)  13.8 (928)  20.8 (1392)  14.7 (983)  13.6 (910)  8.15 ± 0.96  29.57 ± 7.11  130.45 ± 7.88  17.20 ± 2.79  0.54 ± 1.07 | 50.7 (3402)  25.2 (858)  74.8 (2544)  17.5 (596)  19.6 (667)  13.7 (467)  21.1 (719)  15.3 (520)  12.7 (433)  8.14 ± 0.95  29.38 ± 7.17  129.95 ± 7.90  17.22 ± 2.86  0.56 ± 1.07 | 49.3 (3303)  23.1 (764)  76.9 (2539)  18.3 (604)  19.0 (628)  13.9 (458)  20.4 (673)  14.0 (463)  14.4 (477)  8.15 ± 0.98  29.76 ± 7.05  130.97 ± 7.82  17.18 ± 2.71  3.87 ± 1.76 | -  **0.046**  0.235  0.564  **0.002**  **<0.001**  0.834  0.214 |
| **Children’s dietary intake ^a^,** servings/day,  Water  Fruit & vegetables  Sugar-rich foods  Savoury snacks | 3.83 ± 1.73  2.91 ± 1.38  1.21 ± 1.18  0.34 ± 0.48 | 3.80 ± 1.71  2.93 ± 1.37  1.18 ± 1.15  0.33 ± 0.47 | 3.87 ± 1.76  2.88 ± 1.38  1.25 ± 1.21  0.35 ± 0.49 | **0.044**  **0.133**  **<0.001**  **0.004** |
| **Parents** | **All** | **Mothers** | **Fathers** | ***P*** |
| **Demographics**  % (n)  **SES,** % (n)  Low-SES  High-SES | 6705  24.19 (1622)  75.81 (5083) | 88.8 (5952)  23.3 (1383)  76.7 (4569) | 11.2 (753)  31.7 (239)  68.3 (514) | **-**  **<0.001** |
| **Country,** % (n)  Belgium  Bulgaria  Finland  Greece  Hungary  Spain | 17.9 (1200)  19.3 (1295)  13.8 (928)  20.8 (1392)  14.7 (983)  13.6 (910) | 17.2 (1022)  20.7 (1232)  13.5 (806)  20.4 (1213)  14.6 (870)  13.6 (809) | 23.6 (178)  8.4 (63)  15.8 (119)  23.8 (179)  15.0 (113)  13.4(101) | **<0.001** |
| **Age (y)**, mean ± SD  **BMI (Kg/m^2^)**, mean ± SD | 38.54 ± 5.09  24.40 ± 4.54 | 38.18 ± 4.90  24.08 ± 4.53 | 41.35 ± 5.66  26.88 ± 3.75 | **<0.001**  **<0.001** |
| N= 6705, except for savoury snacks, N= 5765. Boldface indicates statistical significance between sexes at p<0.05. Chi-square test was used to test differences by SES for categorical data. U-Mann Whitney tests were performed to test differences by SES in log-transformed continuous variables.  Abbreviations: BMI, body mass index; z-BMI, Body mass index z score according to Cole. et al (2010); y, years; SD, standard deviation.  Fruits & vegetables: fresh or frozen fruit & berries, canned fruit, fresh fruit juices and vegetables.  Sugar-rich foods: Sugar-sweetened beverages (sugar juices and soft drinks) and sweets.  Savoury snacks: salty snacks and fast-food (e.g.1 small hamburger, 1 small bag of chips, 1 slice of pizza)  ^a^ Dietary intake was assessed with a food frequency questionnaire and answers were transformed into servings per day. | | | | |

| **Table S3.** Description of children’s dietary intake (servings/day) according to socioeconomic status by country | | | | | | | |
| --- | --- | --- | --- | --- | --- | --- | --- |
|  | **Total** | **Belgium** | **Bulgaria** | **Finland** | **Greece** | **Hungary** | **Spain** |
| **Water**  **Low-SES**  **High-SES** | 3.78 ± 0.04  3.85 ± 0.02  *p*=0.180 | 2.89 ± 0.10  3.10 ± 0.05  *p*=0.066 | 4.41 ± 0.09  4.12 ± 0.05  ***p*=0.004** | 2.61 ± 0.18  2.43 ± 0.06  *p*=0.354 | 4.48 ± 0.06  4.50 ± 0.05  *p*=0.860 | 3.75 ± 0.09  4.17 ± 0.08  ***p*=0.001** | 4.40 ± 0.32  4.58 ± 0.05  *p*=0.574 |
| **Fruits & vegetables**  **Low-SES**  **High-SES** | 2.82 ± 0.04  2.93 ± 0.02  ***p*=0.010** | 3.10 ± 0.07  3.24 ± 0.04  *p*=0.092 | 3.00 ± 0.08  3.06 ± 0.04  *p*=0.517 | 2.43 ± 0.15  2.75 ± 0.05  ***p*=0.042** | 2.74 ± 0.06  2.90 ± 0.05  ***p*=0.046** | 2.58 ± 0.07  2.54 ± 0.07  *p*=0.639 | 2.70 ± 0.32  2.99 ± 0.05  *p*=0.375 |
| **Sugar-rich foods**  **Low-SES**  **High-SES** | 1.40 ± 0.03  1.16 ± 0.02  ***p<0.001*** | 1.73 ± 0.07  1.54 ± 0.03  ***p*=0.014** | 1.42 ± 0.06  1.18 ± 0.03  ***p<0.001*** | 0.67 ± 0.07  0.69 ± 0.02  *p=0.071* | 0.95 ± 0.03  0.92 ± 0.03  *p=0.411* | 2.19 ± 0.10  1.57 ± 0.09  ***p<0.001*** | 1.31 ± 0.16  0.99 ± 0.02  ***p=0.048*** |
| **Savoury snacks**  **Low-SES**  **High-SES** | 0.42 ± 0.01  0.30 ± 0.01  ***p<0.001*** | 0.34 ± 0.02  0.25 ± 0.01  ***p<0.001*** | 0.55 ± 0.03  0.39 ± 0.02  ***p<0.001*** | 0.22 ± 0.01  0.19 ± 0.004  ***p=0.025*** | 0.29 ± 0.01  0.25 ± 0.01  ***P=0.029*** | 0.66 ± 0.04  0.47 ± 0.04  ***p<0.001*** | **N.A.** |
| N= 6705, except for savoury snacks, N= 5765. ANCOVA adjusted for country, parental BMI, and age sex, and children’s BMI age and sex.  SES, Socioeconomic status was determined by parental education as low (14 or less years of education) or high (14 years of education or more). | | | | | | | |
